# Activity and Synergy of Cu-ATCUN Antimicrobial Peptides

**DOI:** 10.3390/ijms232214151

**Published:** 2022-11-16

**Authors:** Jenna M. Greve, J. A. Cowan

**Affiliations:** Department of Chemistry and Biochemistry, The Ohio State University, 100 West 18th Avenue, Columbus, OH 43210, USA

**Keywords:** synergy, antimicrobial peptides, ATCUN

## Abstract

Antibiotic resistance demands innovative strategies and therapies. The pairs of antimicrobial peptides tested in this work show broad-spectrum synergy and are capable of interacting with diverse bacterial membranes. In most cases, the ATCUN motif enhanced the activity of peptides tested in combination. Our studies also show CP10A to be a multifaceted peptide, displaying both cell membrane and intracellular activity and acting as a chameleon, improving the activity of other peptides as needed. The results of the synergy experiments demonstrate the importance of varied modes of action and how these changes can affect the ability to combat pathogens, while also illustrating the value of the metal-binding domain in enhancing the activity of antimicrobial peptides in combination.

## 1. Introduction

**Figure 1:**
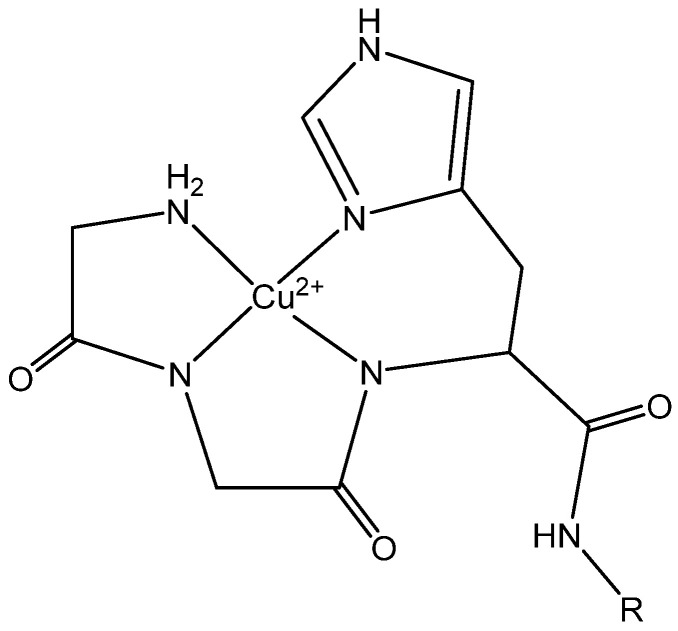
Structural detail of the ATCUN motif at the N-terminus of an AMP (R).

While beneficial to society, the introduction of antibiotics for human health and economics destroyed the equilibrium between antibiotics and antibiotic-resistant microbes. The evolutionary mechanisms of bacteria ensure that persistent efforts to develop and utilize novel therapeutics to fight infection are necessary. Some argue that we are already in a post-antibiotic era and in dire need of innovative therapies. In 2019, while 42 new antibiotics were in development, only 4 new drug applications were in process and only 1 of the 4 functioned in a new class or mechanism of action [1]. However, innovative technologies are under evaluation and include promising results utilizing bacteriophage, antibodies, and potentially vaccines as novel methods of combating antibiotic resistance [2]. Another promising therapeutic approach, the focus of our work, is in the development of catalytically active antimicrobial peptides utilizing the amino-terminal copper and nickel (ATCUN) motif (Figure 1).

Although antimicrobial peptides have been successfully leveraged as antibiotics (e.g., bacitracin), their combination with catalytically active metal-binding domains to promote irreversible chemistry is a unique approach. Antimicrobial peptides are often touted as being more effective at preventing the rise of antimicrobial resistance due to their lack of specificity, and their ubiquity in nature [3,4,5].

**Figure 2:**
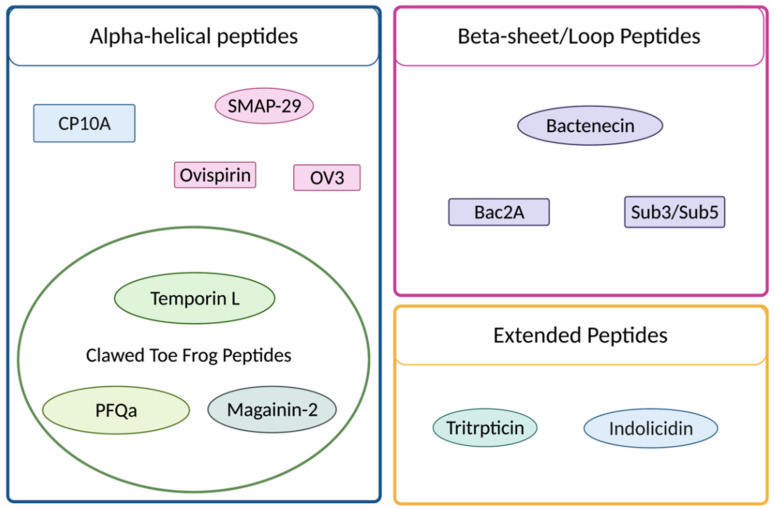
Selected peptides sorted by structure. Ovals represent naturally derived peptides and rectangles represent rationally designed derivatives. Parental and derivative peptides are sorted by color with CP10A being a derivative of indolicidin, Bac2A and Sub3/Sub5 being derivatives of bactenecin and ovispirin and OV3 being derivatives of SMAP-29. The clawed-toe frog peptides are further segregated by a green circle; all other peptides are cathelicidin peptides. Tritrpticin is classified as an extended/ β-turn peptide.

The nonspecific mechanism of AMPs may be considered an advantage; resistance is still a recognized problem and has led to the desire for additional therapeutic approaches [6]. Varying classes of peptides and several families of peptides have exhibited the potential for synergistic interaction, including a variety of cathelicidins of distinct structural classes (e.g., SMAP-29, indolicidin, tritrpticin and bactenecin) and clawed-toe frog peptides (e.g., magainins and temporins), as illustrated in Figure 2. Aside from identifying new antimicrobials, increasingly attractive avenues for exploration include the utility of synergy among current therapeutics and application of multifunctional molecules. Both of these attributes have been discussed in prior publications from this laboratory [7,8]. Synergistic interactions involve the application of drug combinations where the observed effect is greater than the additive contributions from each of the individual molecules. The effect is both facilitated and further amplified in the case of antimicrobial peptides that display more than one mode of action, encompassing both membrane-penetrating and -destabilizing influences, as well as distinct intracellular target(s) that can include mitochondrial, nuclear, Golgi, or other cytosolic molecules.

In this paper, we examine the influence of antimicrobial peptides from the cathelicidin and clawed-toe frog peptide families. We further examine peptide derivatives that have been further modified by the addition of an amino terminal copper/nickel-binding (ATCUN) motif, which has been shown to offer distinct modes of activity to known antimicrobial peptides [7,9]. The antimicrobial and antiviral activities of ATCUN-modified peptides are now extensively documented [7,9,10,11,12,13]. Before proceeding to the results of the investigations, a brief introduction to the cathelicidin and clawed-toe frog derivative peptides used in these studies is provided.

### 1.1. Cathelicidins

Sheep myeloid antimicrobial peptide (SMAP-29) has broad-spectrum activity against a variety of organisms [14]. The structure consists of an alpha-helical N-terminal region, a hinge and then an ordered hydrophobic C-terminal region with the alpha-helicity being elicited upon contact with a membrane [15]. The antimicrobial activity is driven by the N-terminal alpha-helical segment while the cytotoxicity to mammalian cells has been shown to be driven by the hydrophobic region [15,16,17]. In an effort to improve the therapeutic index, ovispirin peptides were developed to improve activity and reduce hemolytic activity [18]. SMAP-29 and its derivatives have been shown to rapidly permeabilize bacterial cells, causing depolarization of the cell membrane and membrane damage, indicating they are bactericidal and not just inhibitory to growth [18,19,20]. MICs for SMAP-29 in a broad range of organisms are potent, with the majority being below 2 µM [19].

Similar to many AMPs, SMAP-29 is membranolytic to eukaryotic cells, though generally at higher concentrations than for bacterial cells. Little evidence has thus far been reported for any SMAP-29 synergistic activity. Mild synergy with lysozyme and lactoferrin was reported in *E. coli* but fell off when tested in other organisms, such as MRSA [21]. The ovispirin derivatives of SMAP-29 consist of linear peptides, generally mimicking the N-terminal region [17,18]. While not identical, the truncated and amino acid substituted derivatives are meant to mimic the activity of SMAP-29, while reducing negative off-target effects. Indolicidin is another cathelicidin, derived from bovine neutrophils, and is one of the smallest cathelicidin peptides at only 13 amino acids. In contrast to SMAP-29, indolicidin does not exhibit a 3D structure, even upon contact with membranes, categorizing it as a linear or extended structure [22]. This class of peptides often exhibits a large fraction of a single amino acid, such as glycine, proline, tryptophan or histidine [23].

Indolicidin contains a higher proportion of tryptophan and proline residues [22]. Other examples include, tritrpticin, lactoferricin and the human histatins [24]. The C-terminus is naturally amidated, which has been shown to reduce hemolytic activity toward mammalian cells [25]. Indolicidin exhibits broad-spectrum antimicrobial activity but is still lytic to human erythrocytes, limiting its therapeutic potential [22,26]. CP10A, an analogue of indolicidin, showed enhanced activity in Gram-positive organisms and an altered secondary structure [27]. CP10A substitutes alanine for proline, allowing an alpha-helical secondary structure to form. Indolicidin has been shown to target proteins and RNA and DNA in the cell, impairing DNA replication and transcription by preventing the unwinding of the double helix [28,29,30]. Targeting of proteins was able to show the inhibition of aminoglycoside phosphotransferase and acetyltransferase in vitro [31]. Like many AMPs, it also disrupts cell membranes [32,33].

Tritrpticin, named for the three Trp residues, is an almost palindromic sequence rich in tryptophan, proline and arginine residues, and is derived from porcine bone marrow [34]. The peptide is unstructured in buffer and adopts a variety of conformations upon interaction with membranes including a β-turn [35]. Like indolicidin, tritrpticin exhibits broad-spectrum activity. Substitution of arginine with lysine in the sequence improved the antimicrobial activity and reduced the lytic activity against human cells [36,37]. While shown to form toroidal pores to disrupt the membrane, it is suspected, though not rigorously confirmed that tritrpticin exhibits activity against intracellular targets much in the same way that indolicidin does [38]. Bactenecin is derived from bovine neutrophils and is a disulfide bonded type 1 beta-turn peptide [39]. Like the others, bactenecin displays broad-spectrum antimicrobial activity but also hemolysis. In an attempt to functionalize the peptide for therapeutic purposes, a variety of derivatives were created, including Bac2A, a linear derivative, and Sub3 and Sub5 [40]. Unlike some other derivatives, (e.g., indolicidin and CP10A), the beta-turn structure is maintained in the Sub3 and Sub5 derivatives. Sub3 and Sub5 have more potent activity than their parental bactenecin with more limited hemolysis [41]. The broad-spectrum activity proceeds without significant membrane disruption. They have been shown to interact with the cell surface but ultimately accumulate intracellularly and are able to inhibit ATP-dependent enzymes by interacting with ATP [42,43,44].

### 1.2. Clawed-Toe Frog Peptides

There are 33 species of clawed frogs, among five genera belonging to the family Pipidae, which produce a variety of potent antimicrobial peptides. The magainin peptides are derived from the African clawed-toe frog *Xenopus laevis* [45]. The two most studied magainin peptides, magainin 1 and magainin 2, are amphipathic-helix-forming structures that disrupt membranes via toroidal pore formation [46]. Another peptide, PFQα, is derived from the skin secretion of the African frog *Silurana epitropicalis,* which is a species phylogenetically related to *Xenopus laevis*, from which the magainins are derived [47]. There is phylogenetic evidence that *Xenopus* and *Silurana* are a united subfamily (*Xenopodinae*) [48]. While magainin-2 has no detectable antimicrobial activity against MRSA, PFQα was shown to have low micromolar activity against MRSA [45]. Temporins represent another family of peptides derived from the skin of a different species, the European frog *R. temporaria,* and represent one of the largest families of frog peptides with over 100 isoforms [49]. They are linear peptides with 8–17 residues and form amphipathic alpha helices in membrane mimetic environments [50]. They notably have a lower positive charge than most other antimicrobial peptides, ranging from 0 to +3, with most having a single basic residue [51]. The lack of a positive charge has been linked to the family being effective against Gram-positive organisms but lacking activity against Gram-negative organisms [49,52]. One notable exception is the highly studied TempL, which has an additional positive charge and increased activity against Gram-negative organisms [53]. Like many AMPs, TempL exhibits cytotoxicity toward human cells at its biocidal concentrations. No intracellular targets have been defined thus far for temporins, but the short length of these peptides precludes them from pore formation in membranes. Either a synergistic mechanism or carpet-like model can describe their ability to disrupt membranes at higher concentrations, but their ability to disrupt biochemical processes intracellularly, leading to cell death at lower concentrations, cannot be ruled out.

Each of these peptide families exhibit a range of activities that underlie their potential as therapeutics. The varying modes of action, addition of the ATCUN motif and combination therapy are likely to attenuate the development of resistance. A significant amount of work has been completed in our laboratory characterizing the biochemical properties of the catalytically active version of these peptides [7,8]. This report describes work to further characterize the therapeutic potential of a wide variety of peptides and how their mechanisms may act together to further potentiate antibiotic activity.

## 2. Results

### 2.1. MIC Activity

The minimum inhibitory concentration of a peptide is the concentration at which no observable bacterial growth can be determined by visual inspection. MIC values are useful for determining the potential of a test article as a therapeutic, as well as in comparison to other test articles. A variety of antimicrobial peptides were tested for activity against an array of clinically relevant bacterial strains. The sequences of SMAP-29 derivatives and their key features can be seen in Table 1, with peptides being named in reference to how they were originally published. Peptides OV3-00 and OV3-01 were utilized in this study. Because the template (or parental) peptide, SMAP-29, derived from sheep leukocytes, is hemolytic to human red blood cells, efforts were made to enhance the therapeutic index of the peptide leading to the creation of OV and OV3 [14,19]. Various derivatives were made and OV3 came to represent two distinct peptides in the literature: the peptide labeled OV3 and a peptide with an additional c-terminal YG, herein labeled OV3-00. Previous work in our laboratory utilized OV3 for the design and synthesis of ATCUN domain peptides, but it lacks a tryptophan or tyrosine residue that allows for more ready quantification of the peptide concentration by the extinction coefficient, leading to a less accurate, more difficult, concentration analysis by mass spectrometry [8]. To ease quantification and facilitate studies on the OV3 series in our laboratory, and compare the effect of the added YG, additional work was pursued using the longer OV3-00 and OV3-01 derivatives.

Comparison of MIC values among the OV3 derivatives shows that the addition of the YG hook at the C-terminal end of the peptide drastically improved the activity of the parental OV3-00 peptide as compared to the OV3 parental peptide in *Escherichia coli* 25922 (Table 2). As in previous work conducted in our laboratory, the addition of the GGH motif did not seem to enhance the antimicrobial activity of the peptide but does not abrogate the activity either [7,8,11]. Of interest is the observation that no peptide across the tested strains retained improved activity over the others. While the YG derivatives had equal or better activity than the OV3 peptide, several of the peptides with increased alpha-helicity performed at equivalent or only slightly improved activities relative to OV3-00 and OV3-01.

A variety of other peptides (primarily derived from the clawed-toe frog family) were also tested for antimicrobial activity to determine potential candidates for synergy testing (Table 3). Similar to the SMAP-29 series of peptides, many of the peptides with the added ATCUN motif saw little enhancement or a detrimental effect to the antimicrobial activity of the peptide (Table 4). One exception was the copper-binding derivatives of tritrpticin and indolicidin, which seemed to systematically lose antimicrobial activity relative to the activity of the parental peptide, with MIC values diminishing 4-fold for the tested bacterial strains. While there was a systematic decrease in the ability of tritrpticin and indolicidin derivatives to inhibit activity across bacterial strains, most peptides saw variation in the activity across different strains of bacteria that tracked similarly between the parental and ATCUN motif peptides. MICs ranged from low µM to greater than 128 µM, with the Sub5 peptides previously characterized in our laboratory having the lowest MIC values [7]. One peptide, PFQα-01, showed poor activity, only exhibiting detectable activity in MRSA (MIC 16 µM) and in *Acinetobacter*, with an MIC of 128 µM.

### 2.2. FIC Activity

Determining MIC values informed decisions on testing peptides in combination. Fractional inhibitory concentrations determine the ability of test articles to act in concert against a pathogen. FIC determination is experimentally similar to MIC determination, except one test article is diluted down the wells and another is diluted across the wells of the 96-well plate in order to test the synergism of two peptides. The MIC can be determined in the bottom row and the 10th column for each of the respective peptides, and the FIC well is the well containing the lowest concentration of each peptide that shows no visible growth, which, like the MIC assay, can be determined by visual inspection. Synergy is defined as an FIC of less than or equal to 0.5, meaning there is a four-fold reduction in the concentration of each peptide required for inhibition. Any number above 4 indicates a deleterious interaction that reduces the effectiveness of each peptide, and a value between 0.5 and 1 can be considered additive (Equation (2)).

As the FIC assay is significantly more resource-intensive than MIC assays, multiple combinations of peptides were first tested in duplicate in *E. coli* 25922 for potential synergistic interactions (SM1 and SM2). Peptides with MIC values in *E. coli* less than or equal to 32 µM were considered. CP10A, TempL, CP29-01, OV3 derivatives, Mag2 derivatives, Sub5 derivatives, tritrpticin derivatives and indolicidin derivatives were tested in a variety of combinations (see Table 3 for a full list of peptides and sequences). Interactions between peptides that were additive or indifferent can be seen in Table S1 for interactions with OV3 peptides and Table S2 for interactions with CP10A and TempL peptides. To best characterize how synergistic interactions occurred, multiple types of peptides were tested against each other. Interactions that were additive or indifferent included OV3-00 with the tritrpticin and indolicidin ATCUN variants, which all adopt wedge-shaped or beta-turn structures. The OV3-00 parental, OV3-01 and Mag2-01 ATCUN derivative also failed to produce any synergistic interaction.

Previous work in our laboratory has reported a synergistic interaction between CP10A and Sub5 [11], so it was of continued interest to determine other potential synergistic interactions between CP10A and varying peptide derivatives. Like OV3-00, CP10A showed additive or indifferent activity with the wedge shaped tritrpticin and indolicidin derivatives in *E. coli* 25922. TempL showed additive activity with the hybrid CP29-01 peptide and two of the four indolicidin tritrpticin derivatives (Table S2).

**Figure 3:**
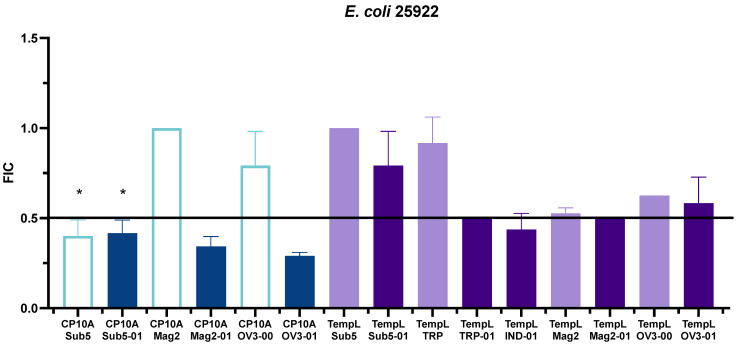
Representation of FIC values for interactions of CP10A (blue) and TempL (purple) in *E. coli* 25922. ATCUN-containing derivatives are represented as dark blue or dark purple, and parental peptides are represented as light blue or light purple. The line represents the threshold for synergy at 0.5. Starred data (*) are previously reported data from reference [11] for comparison.

FIC values that showed promising synergy in initial *E. coli* 25922 testing were repeated in triplicate and further investigated. Synergistic interactions in *E. coli* 25922 included several ATCUN derivative peptides in combination with CP10A and TempL (Figure 3, Table S3). The ATCUN-containing peptides, including Sub5-01 [11], Mag2-01 and OV3-01, all displayed strong synergistic interactions with CP10A. TempL also displayed synergy with the ATCUN derivatives TRP-01, IND-01 and Mag2-01, though with synergy displaying an FIC at the border of synergy and additive. The TempL/OV3-01 and TempL/Sub5-01 interactions that were potentially expected to be synergistic were found to be additive in nature. The FIC was also determined for the synergistic peptide template peptide (non-ATCUN-containing) to determine if the ATCUN motif played a role in the synergistic interaction. With the exception of the CP10A/Sub5 interaction, all of the parental peptides showed additive or indifferent, not synergistic, activity with the tested CP10A and TempL (Figure 3, Table S4). The CP10A Mag2-00 and OV3-00 interactions had much larger FIC values than their synergistic ATCUN counterparts. The value for TempL/Tritrpticin ATCUN derivatives were at the highest definable limit of synergy but their parental interaction was also much higher (Figure 3, Table S4). While the TempL/Mag2-01 interaction was synergistic, it also lay on the border of the highest definable limit of synergy. The TempL/Mag2-00 FIC value was much closer to the ATCUN motif derivative than the others with an FIC value of 0.53.

**Figure 4:**
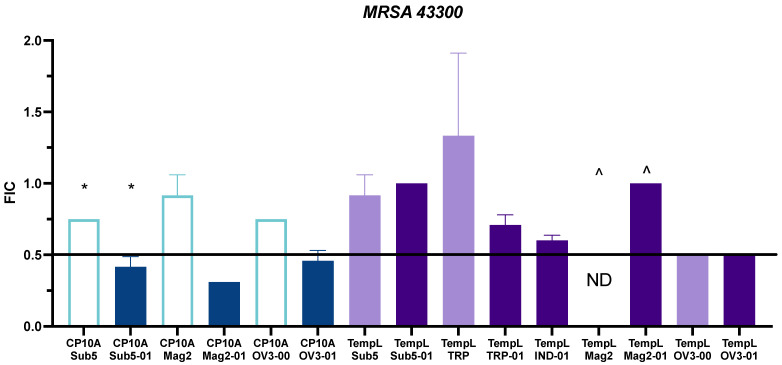
Representation of FIC values for interactions of CP10A (blue) and TempL (purple) in MRSA 43300. ATCUN-containing derivatives are represented as dark blue or dark purple and parental peptides are represented as light blue or light purple. The line at 0.5 represents the threshold for synergy. For data marked (^), Mag2-01 had no detectable MIC in MRSA 43300, and the FIC value was calculated from the lowest detectable concentration on the FIC plate. Mag2 parental activity was not determined (ND). Error is reported as the standard deviation of the mean. Starred data (*) are previously reported data from reference [11] for comparison.

To further examine the synergism of the tested peptides, FIC values were also tested in MRSA 43300 (Figure 4). Values for the ATCUN derivatives and parental derivatives can be seen in Table S6. For CP10A, interactions with Sub5-01, Mag2-01 and OV3-01 retained synergy. Unlike in the case of *E. coli* 25922, CP10A/Sub5 was not synergistic, but additive. This held for the CP10A/Mag2-00 and CP10A/OV3-00 interactions as well, showing a persistent pattern across the two strains of the ATCUN motif delivering a synergistic interaction with CP10A. The TempL interactions were less promising, with the ATCUN derivative synergy falling off to additive and indifferent, with the exception of the TempL/OV3 interaction, where both peptides, the parental and ATCUN derivative, displayed an equal synergy of 0.5.

Historically, magainin-2 has not exhibited antimicrobial activity against MRSA [57]. The TempL/Mag2-01 interaction indicates as much (Figure 4, Table S5), with the FIC plate showing an indifferent FIC value of 1. The activity of TempL and Mag2-00 was not determined for this reason. One major finding of this work is that CP10A restores Mag2-00 and Mag2-01 activity against MRSA, giving detectable synergistic and additive values. While the MIC value was determined to be greater than 128 µM, the FIC was calculated using the highest concentration of peptide utilized on the plate, which represents the highest threshold for the calculation. Not only did CP10A recover activity in Mag2-01, reducing the required peptide to 4 µM, but it also acted synergistically with a strong FIC value of ≤0.31. The CP10A/Mag2-00 interaction was not synergistic, but it did recover Mag2 activity by at least four-fold to between 16 and 32 µM on the plate in combination with CP10A (Table S6).

**Figure 5:**
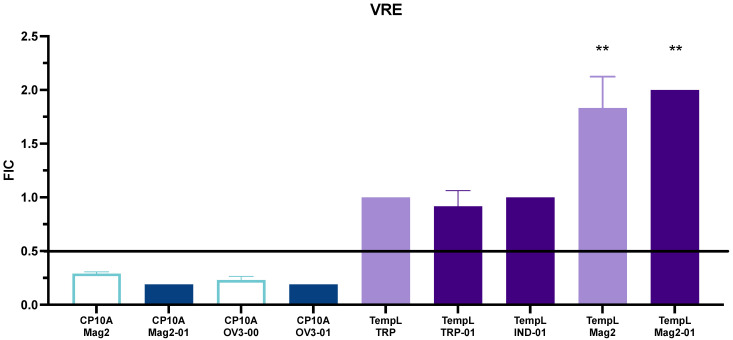
Representation of FIC values for interactions of CP10A (blue) and TempL (purple) in VRE. ATCUN-containing derivatives are represented as dark blue or dark purple, and parental peptides are represented as light blue or light purple. The line at 0.5 represents the threshold for synergy. Data marked (**) indicate biofilm formation, and no detectable MIC was determined for Mag2-01 below 32 µM. Error is reported as the standard deviation of the mean.

Two additional strains were tested, for a total of two Gram-positive and two Gram-negative organisms. In addition to *E. coli* 25922 and MRSA 43300, the clinically relevant *A. baumannii* BAA747 and vancomycin-resistant *E. faecium* 700221 (VRE) were utilized to screen for synergy. As in the case of MRSA, the TempL/TRP-01 and TempL/IND-01 combinations maintained only additive interactions (Tables S9 and S10), showing synergy only in the case of *E coli*. The TempL/Mag2-01 combination was found to be synergistic in the Gram-negative *Acinetobacter*, but no synergy was observed in the Gram-positive VRE strain. The CP10A/Sub5 interactions were both additive, with the ATCUN domain providing no additional synergy in *Acinetobacter* (Tables S9 and S10). CP10A/Mag2-01 and CP10A/OV3-01 were also synergistic in *Acinetobacter*, improving the activity over their additive parental peptides. While both ATCUN and parental peptides showed synergy in VRE, the CP10A/OV3-01 and Mag2-01 interactions exhibited enhanced synergy over their non-ATCUN counterparts (Figure 5, Tables S7 and S8).

## 3. Discussion

### 3.1. Role of MICs in Determining Therapeutic Potential

Testing the minimum inhibitory concentration of a potential antibiotic is a key step in determining the therapeutic viability of a compound. A significant amount of work has been carried out previously in our laboratory, and others, to determine the inhibitory concentrations and biochemical characteristics of a variety of peptides. Notably, MICs can be difficult to compare due to variations in experimental conditions and interlaboratory variations that are known to impact the activity of antimicrobial compounds [57]. It is important to consider experimental conditions when comparing MIC values between publications. Elucidating the ability of a variety of AMPs, both ATCUN-containing and parental, to act effectively on not only test strains but also clinically relevant isolates is important in determining the value of a potential therapy. SMAP-29 and its derived peptides all exhibit broad-spectrum low µM activity against a variety of isolates [8,14,17].

The effect of the C-terminal YG has been shown in the parental OV3 to be important for membrane interaction and may confer some of the improved antimicrobial activity of OV3-00 and OV3-01 over the previously studied OV3 [18]. SMAP-29 and ovispirin are notoriously hemolytic to human cells, which has led to vigorous efforts to determine the role of AMP structure in antimicrobial activity and cytotoxicity [17]. While the increased alpha helicity of the Cu-2 and Cu-4 peptides previously studied in our laboratory [8] can account for the improved antimicrobial activity of the ATCUN-OV3 derivatives, they retained reduced cytotoxicity relative to SMAP-29 [8]. These small variations in structure, the C-terminal YG and the increased helicity arising from GGC, improve activity relative to WT, although in different ways, and show the importance of minor changes to these AMPs. Previous work in our laboratory has also determined alternative functional mechanisms for ATCUN-containing peptides, including lipid oxidation, enhanced membrane permeabilization, increased ROS generation and nuclease activity [7,8,13]. The MIC values for a variety of other peptides were also determined to screen for potential AMPs to test for synergy. While the values produced were uninspiring as true lead hits for potential therapeutics, all showed a broad-spectrum activity against a variety of organisms except PFQα-01. PFQα is an AMP shown to have activity only in MRSA [47] and was screened with the ATCUN motif to determine if additional activity against other strains could be conferred. While it did gain 128 µM activity against Acinetobacter, it failed to produce detectable values in any of the other tested strains, including a different strain of S. aureus. Studying the mechanism of action of PFQα against MRSA may lead to improved rational design decisions in targeting the pathogen in the future.

To screen for synergy between peptides pulled down together from the frog species Rana dybowskii [58], dybowskin-1 and dybowskin-2 ATCUN derivatives were synthesized, but the ATCUN motif eradicated any antimicrobial activity up to 128 µM. These peptides exhibit the complicated task of enhancing activity, and like the OV3 examples, they show the significant effects small changes can have in peptide activity and action. While the MIC values provide insight into the therapeutic viability, and comparison of peptide variations can be informative, for the currently investigated peptides these values serve as an intermediate step to screen for suitable synergy candidates.

### 3.2. Synergy

While standards in the literature give hard cutoffs for FIC values, it is best to think of the potential for peptides to act in concert on a spectrum, with lower FIC values being more desirable than their still synergistic counterparts that reside at the top of the FIC synergy cutoff. With that idea, even among additive interactions, there may be therapeutic value in treating infections. Oxidation of the lipid bilayer has been shown to enhance the activity of Temporin B and L by improving intercalation in model membranes [59]. This idea, combined with the previously reported ability of ATCUN-domain-containing AMPs to oxidize lipid bilayers [7,8], led to the idea of testing ATCUN AMPs in conjunction with TempL, and potentially other antimicrobial peptides.

**Figure 6:**
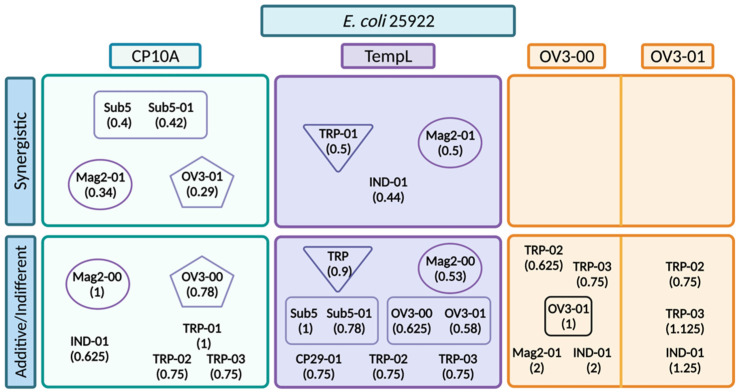
Summary of peptide interactions in *E. coli* 25922 from data presented in Results and Supplemental Material. Synergistic (or non-synergistic) interactions for CP10A, TempL, OV3-00 and OV3-01 with other peptides are grouped as shown. Parental and ATCUN derivatives are outlined in the same shape with the average FIC value shown in parentheses.

**Figure 7:**
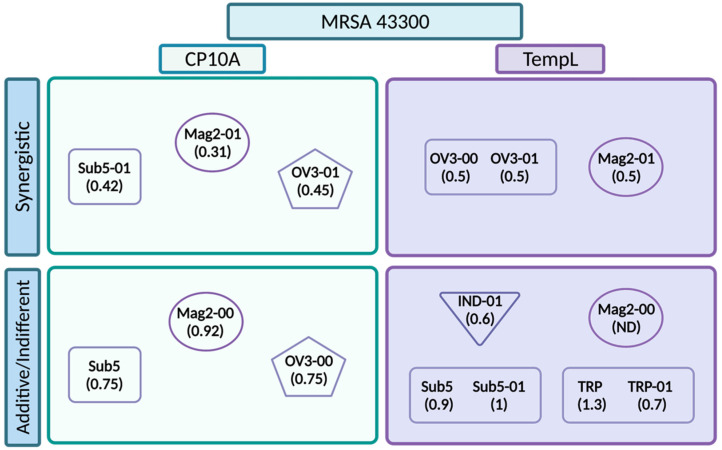
Summary of peptide interactions in MRSA 43300 from data presented in Results and Supplemental Material. Synergistic (or non-synergistic) interactions for CP10A and TempL with other peptides are grouped as shown. Parental and ATCUN derivatives are outlined in the same shape with the average FIC value shown in parentheses.

Many peptides interact synergistically in *E. coli*; however, their activity quickly drops off in other organisms, showing the difficulty of achieving broad-spectrum activity [60,61,62]. While the TempL interactions initiated this inquiry, they were not the most promising synergistic interactions. The synergistic interaction with the wedge and extended TRP-01 and IND-01 peptides dropped off in organisms beyond *E. coli*, as in other referenced examples (Figure 6). TempL is not long enough to span the membrane and is generally thought to interact with membranes via a carpet model [50], while the non-helical TRP-01 and IND-01 are derived from peptides that are thought to act more like cell-penetrating peptides. The ATCUN OV3 peptides are known to oxidize lipid bilayers [8]; however, their action did not seem to cause synergy in *E. coli* (Figure 6). They were effective in MRSA 43300 (Figure 7), complicating the previously reported literature as a viable practical application for temporin. The cause of the synergy in MRSA has also come into question, as the ATCUN and parental OV3 derivatives displayed equal synergy with TempL (Figure 7). There was no enhancement with the ATCUN derivative, unlike in other pairs. This lack of improved synergy with the ATCUN motif indicates that the synergistic interaction does not depend on the lipid oxidation mechanism present in the ATCUN-containing peptide but may be a result of other preferential membrane activity. With the exception of TempL/OV3 and TempL/OV3-01 in MRSA, the addition of the ATCUN motif in each tested set was observed to enhance the activity of peptides in combination across all strains of bacteria examined. While not all pairs became synergistic, their additive FIC improved, meaning less of each peptide was required to achieve the same inhibitory result. This is attributable to the ATCUN motif’s ability to stress cells, the creation of ROS that dysregulate cellular processes and its ability to further disrupt cell membranes [7,8,10,11,12,63,64].

While CP10A was not synergistic with any of the extended/beta-turn tested indolicidin/ tritrpticin ATCUN derivatives, it did exhibit synergy with the loop peptide Sub5-01 [11]. CP10A is derived from indolicidin, and it is expected that peptides with similar mechanisms would not exhibit synergy. It also follows that neither CP10A nor indolicidin/tritrpticin aids the other’s ability to cross the cell membrane to target intracellular targets. None of the tested template/ATCUN-derivative pairs exhibited synergy. Indolicidin and the template peptide of Sub5, bactenecin, are both cathelicidins from *Bos taurus.* Bactenecin and indolicidin were previously found to be synergistic against *E. coli*, and several subsequent studies explored their mechanism of action, which involves disruption of the membrane and intracellular targets such as DNA [29,32,40,65,66,67]. This previous relationship of synergy between the template peptides may also explain the synergy of the beta-turn Sub5 and Sub5-01 peptides, but lack of synergy with other beta-turn and extended peptides with CP10A. While Sub5 was synergistic in *E. coli*, the synergy was lost in subsequently tested strains. Sub5-01, however, retained synergy in other tested strains. In our previously published work, we showed an enhanced ROS generation, increased DNA damage via TUNEL assays and co-localization of the CP10A/Sub5-01 synergistic pairs, providing a potential mechanism of synergy for these pairs [11].

In the four tested strains, ATCUN derivatives of OV3 and magainin-2 enhanced the level of activity from additive to synergistic, except in the case of *E. faecium*, where both the template and ATCUN derivatives were found to be synergistic. The phospholipid bilayer of the *E. faecium* membrane contains greater than 20% of the positively charged lysyl phosphatidylglycerol, which is a departure from other tested strains [68]. The difference in the *E. faecium* membrane composition may account for the synergy in the parental peptides due to the creation of strain in the lipid bilayer after cationic AMPs are sequestered from the cationic lipid composition. This creates a phase-boundary defect, increasing permeability and leading to enhanced antimicrobial activity. The increased cationicity and helicity of the magainin-2 and OV3 may explain their enhanced activity over the less charged TempL, and beta-sheet and unstructured Sub5, indolicidin and tritrpticin peptides. The enhanced synergy in the ATCUN derivatives is potentially attributable to the aforementioned mechanism in addition to the known biochemical capabilities of the ATCUN domain and intracellular targets.

The interactions of magainin-2 and its ATCUN derivative were particularly interesting. The divide in Gram-positive vs. Gram-negative activity with TempL shows the importance of membrane composition in these interactions. Gram-negative bacteria are thought to be more protective against cationic AMPs because of their robust LPS structure that can both attract and capture AMPs before they reach the cytoplasmic membrane [68,69,70]. LPS can induce oligomerization of peptides on the outer membrane, preventing translocation and activity [71,72,73]. TempL promotes disaggregation of other temporin family peptides in the outer membrane LPS, acting synergistically to allow their translocation to be active on the cytoplasmic membrane [74,75]. Magainin-2 has a characterized, strong interaction with LPS [76,77,78]. It follows that TempL is capable of promoting the passage of magainin-2 across the outer membrane, allowing it to more readily reach the cytoplasmic membrane, where its activity promotes cell death. With this hypothesis in mind, while the ATCUN derivative confers synergy and the template does not, the difference between the two is not robust. In this case, the ATCUN domain may not contribute to the synergistic interaction because of its metal-binding catalytic properties but because of a more nuanced structural difference. This also explains the lack of synergy, or enhancement in synergy, in the Gram-positive organisms.

While TempL failed to improve magainin-2 activity in Gram-positive organisms, the CP10A/Mag2-01 interactions did see an enhancement in activity. Mag2 lacked activity in MRSA as reported in a range of publications, and we also showed a lack of an MIC below 128 µM. When tested in combination with CP10A, not only did the concentration of Mag2-01 required to inhibit MRSA decrease; it also became “synergistic” with CP10A. While based on the technical definition, because we did not have a detectable MIC on the plate, it is difficult to ascribe this term, the activity was so drastically enhanced that we feel it is appropriate to utilize the term. The recovery of Mag2 activity and synergy of Mag2-01 activity are potentially important interactions to study to determine how activity in AMPs can be recovered. Without further biochemical data, it is difficult to comment with certainty on the potential mechanism of the pair, but the phenomenon is a significant and interesting finding.

As a whole, synergistic pairs included a peptide with a known or suspected intracellular target. Model membrane studies and bactericidal studies have shown increasing membrane permeabilization with low amounts of detergent, and improved antimicrobial activity of AMPs with intracellular targets [79,80,81]. The idea that membrane-disrupting peptides improve the activity of intracellular-acting peptides is intuitive and holds in some cases based on the synergy experiments of this chapter, but the interactions are clearly more complicated than membrane disruption plus the intracellular target.

### 3.3. AR Resistance

Canonical antibiotics have been shown to increase the instances of horizontal gene transfer (HGT) [82]. AMPs likely increase HGT as well, as membrane permeabilization leads to the leaking of transposons, eDNA, etc., which allows for increased uptake. The nuclease-targeting abilities of Cu-AMPS, such as the ATCUN-OV3 and ATCUN-Sub5 derivatives, provide an important new mechanism for slowing the spread of antimicrobial resistance. It has also been shown that antibiotics increase the mutation rate, i.e., random mutagenesis, leading to development of resistance [83]. The mutation rate can be measured to determine if an increased number of mutations are occurring that lead to phenotypic resistance to antibiotics after passaging in tolerated doses. It has been shown that increasing concentrations of antibiotics lead to an increase in mutation rate [84]. Induced resistance in MRSA has also been shown with a variety of peptides at sub-lethal dosing after passaging. The MRSA membrane exhibits a reduced negative charge to evade such peptides, but antimicrobial activity was restored, though less potent, after removing selective pressure for several passages. Resistance resumed upon selective pressure again [79]. While it is seemingly intuitive that repeated high exposure to antibiotic induces resistance over time, we can conclude that reducing dosing can reduce mutation and synergistic peptide interactions, and potentially even additive interactions not only become beneficial for cytotoxicity, but also in preventing AR development. Because of this, the reduction in the necessary AMP concentration shown herein is promising as a strategy to combat AMP resistance. The more modes of action an antibiotic possesses, the more difficult it is to destroy its activity, which is another promising feature of ATCUN-containing AMPs.

### 3.4. Conclusions

The synergistic pairs tested in this work show broad-spectrum synergy, being capable of interacting with diverse bacterial membranes. This provides a starting model for effectiveness and activity, but proving in vivo viability is paramount to furthering development of these molecules. In addition, it is necessary to probe the likelihood of these peptides to select for, or induce, antibiotic resistance. It will also be important to test the hemolytic effects on mammalian cells of these peptides at higher concentrations. While their hemolytic activity against mammalian cells may be higher than their FIC concentrations when dosing individually, it will be important to determine if a synergistic effect also exists in mammalian cells. Each of the synergistic pairs in our data has a membrane-targeting peptide and at least one of the two also has a known or highly likely intracellular target. Though not all combinations of a membrane + intracellular target are synergistic (some are just additive), all the combinations that were tested did follow the trend. In addition, the ATCUN motif enhanced the activity of all peptides tested in combination, except the OV3/TempL pair in MRSA. This work also exhibits CP10A as a multifaceted peptide, displaying both cellular membrane and intracellular activity, acting as a chameleon and improving the activity of other peptides as needed. While the MIC values did not drastically improve in our ATCUN derivatives, the varied mechanisms, as illustrated by previous work in our laboratory [7,8,11], and the results of the synergy experiments described here, demonstrate the importance of varied modes of action and how these changes can affect the ability to combat pathogens. These results highlight the value of the metal-binding domain in enhancing the activity of antimicrobial peptides when used in combination.

## 4. Materials and Methods

### 4.1. Peptide Synthesis

Peptides were synthesized utilizing Standard Fmoc SPPS methods [85]. The C-termini of all peptides were amidated to reduce the negative charge using a rink amide MBHA resin. Peptides were deprotected and cleaved from the resin using a cleavage cocktail of 95% trifluoracetic acid (TFA), 2.5% H_2_O and 1.25% ethane dithiol and 1.25% thionanisole, which act as radical scavengers. After synthesis, peptides were purified on a C18 column by reverse-phase HPLC, and purity was checked on a Bruker flex ESI-TOF HRMS.

### 4.2. Minimum Inhibitory Concentrations

Minimum inhibitory concentrations (MICs) of test molecules were established by broth dilution assays. Mid-log phase bacterial cultures of a variety of clinically important organisms were diluted and incubated with a serial dilution of each peptide, starting at 128 µM. The concentration of peptide at which no visible growth occurred was marked as the MIC. Bacterial strains were incubated in Mueller–Hinton Broth (MHB) at 37 degrees Celsius until the OD_600_ reached between 0.8 and 1. The resulting culture was subsequently diluted 100× into 96-well plates with the test articles. Positive and negative controls containing only MHB incubated with bacteria and MHB, respectively, were also included on the plate. Each 96-well plate was incubated for 16–20 h, and the MIC was determined by visual inspection. To determine the colony-forming units (CFUs) present in each assay, 100 µL of the 100× diluted culture was further diluted by 1000× and plated on agar plates and determined by use of Equation (1). The colonies were counted to ensure that the CFU value was between 10^4^ and 10^5^.

CFU/mL = (mean # of colonies/0.1 mL) (1000 (dilution factor))(1)

### 4.3. Fractional Inhibitory Concentrations

Fractional inhibitory concentrations (FICs) were determined in a manner similar to MIC determinations but with serial dilutions of two peptides running both horizontally and vertically across the 96-well plate. Determination of the FIC can be seen in Equation (2), where FIC is the fractional inhibitory concentration, [A] is the concentration of substrate A at MIC in combination, [B] is the concentration of substrate B at MIC in combination, and MIC_A_ and MIC_B_ are the MIC of substrate A and substrate B, respectively. FIC = fractional inhibitory concentration, [A] = concentration of A at MIC in combination, [B] = concentration of B at MIC in combination, $MIC_{A}$ = minimum inhibitory concentration of A, and $MIC_{B}$ = minimum inhibitory concentration of B. When FIC ≤ 0.5 the results are synergistic. If FIC > 4, the results are antagonistic, and when FIC > 0.5, the results are indifferent or additive.

(2) $$FIC=\frac{\left[A\right]}{MIC_{A}}+\;\frac{\left[B\right]}{MIC_{B}}$$

## Figures and Tables

**Table 1:** SMAP-29 derivatives used in this study and others with sequences and defining characteristics. ^a^ Estimated charge calculated from Innovagen’s peptide property calculator. ^b^ The helical activity of peptides as previously reported in the referenced manuscripts. ND: not determined.

Peptide	Sequence	AA	Charge pH 7 ^a^	% Helical (SDS) ^b^
SMAP-29	RGLRRLGRKIAHGVKKYGPTVLRIIRIAG	29	10.1	58% [19]
OV	KNLRRIIRKIIHIIKKYGPTILRIIRIIG	29	10.1	86% [17]
OV3	IRRIIRKIIHIIKK-NH_2_	14	7.1	38% [8]
OV3-00	IRRIIRKIIHIIKKYG-NH_2_	16	7.1	ND
OV3-01	GGHIRRIIRKIIHIIKKYG-NH_2_	19	7.2	ND
Cu-1	GGHGIRRIIRKIIHIKK-NH_2_	18	7.2	14% [8]
Cu-2	GGHGIRRIIRKIIHIIKKGGC-NH_2_	21	7.1	56% [8]
Cu-3	GGHIRRIIRKIIHIIKK-NH_2_	17	7.2	35% [8]
Cu-4	GGHIRRIIRKIIHIIKKGGC-NH_2_	20	7.1	64% [8]

**Table 2:** MIC Values (µM) for SMAP-29 Derivatives. * Previously reported data from reference [8] r comparison.

MIC (µM)	Gram Stain	GGH	OV3-00	OV3-01	OV3 *	Cu-1 *	Cu-2 *	Cu-3 *	Cu-4 *
*E. coli* 25922	(-)	>128	2	4	>16	>16	3	>16	1.3
*A. baumannii* BAA747	(-)	>128	4	8	1	2.7	9.3	5.3	10.7
*P. aeruginosa* (27583)	(-)	>128	16	16	16	>16	12	>16	4
*K. pneumoniae* 700603	(-)	>128	4	16	>16	16	>16	16	8
*S. aureus* 43300 (MRSA)	(+)	>128	8	16	>16	16	4	16	1.5
*E. faecium* 700221 (VRE)	(+)	>128	4	4	16	16	8	8	3.3

**Table 3:** Clawed-toe peptides and sequences utilized in this study and others with sequences and defining characteristics. * Estimated charge calculated from Innovagen’s peptide property calculator.

Peptide	Sequence	AA	Charge pH 7 *	Structure
Mag2	GIGKFLHSAKKWGKAFVGEIMNS-NH_2_	23	4.1	helix [54]
Mag2-01	GGHGIGKFLHSAKKWGKAFVGEIMNS-NH_2_	26	4.2	helix
CP29	KWKSFIKKLTTAVKKVLTTGLPALIS-NH_2_	26	7	helix [27]
CP29-01	GGHKWKSFIKKLTTAVKKVLTTGLPALIS-NH_2_	29	7.1	helix
TRP	VRRFPWWWPFLRR-NH_2_	13	5	β-turn [35]
TRP-01	GGHVRRFPWWWPFLRRGGC-NH_2_	19	5	β-turn [55]
TRP-02	GGHGVRRFPWWWPFLRR-NH_2_	17	5	β-turn [55]
TRP-03	GGHGVRRFPWWWPFLRRGGC-NH_2_	20	5	β-turn [55]
IND-01	GGHILPWKWPWWPWRRGGC-NH_2_	19	4	extended [55]
CP10A	GILAWKWAWWAWRR-NH_2_	14	4	helix [56]
TempL	FVQWFSKFLGRIL-NH_2_	13	3	helix [50]
PFQα	FLGALLGPLMNLLQ-NH_2_	14	1	helix [47]
PFQα-01	GGHYLGALLGPLMNLLQ-NH_2_	17	1.1	helix
Sub5	RRWKIVVIRWRR-NH_2_	12	7	β-loop [7]
Sub5-01	GGHRRWKIVVIRWRR-NH_2_	15	7.1	β-loop [7]

**Table 4:** MIC values for antimicrobial peptides utilized in this study. MIC values for a variety of the presented peptides have been previously determined in alternative conditions; values here are the result of experimental efforts to determine MICs under the conditions utilized for further study of FIC potential, with the exception of Sub5 and Sub5-01, previously characterized in our laboratory, which have been taken from reference [7].

MIC (μM)	Gram Stain	GGH	CP29-01	Mag2	Mag2-01	TRP	IND-01	TRP-01	TempL	PFQα-01	CP10A	Sub5	Sub5-01
*E. coli* 25922	(-)	>128	8	8	16	8	32	32	4	>128	16	3.3	2.7
*A. baumannii* BAA747	(-)	>128	1	8	8	8	32	16	4	128	8	0.8	1
*P. aeruginosa* 27583	(-)	>128	2	32	64	16	>64	64	32	>128	64	4	2
*K. pneumoniae* 700603	(-)	>128	8	64	64	32	>64	32	16	>128	32	1.7	2
*S. aureus* 43300 (MRSA)	(+)	>128	16	>128	>128	8	64	32	4	16	4	2	1
*E. faecium* 700221 (VRE)	(+)	>128	4	16	32	2	8	8	8	>128	8	0.5	0.4

## Data Availability

Data are provided in the text and Supplementary Material.

## Supplementary Materials

The following supporting information can be downloaded at: https://www.mdpi.com/article/10.3390/ijms232214151/s1, additional tables of FIC results.
