# Supplementary material for: Activity and Synergy of Cu-ATCUN Antimicrobial Peptides
Source: Int J Mol Sci. 2022 Nov 16;23(22):14151. doi: 10.3390/ijms232214151 (PMC9692552; doi:10.3390/ijms232214151)
Supplement: Supplementary file 1 [file ijms-23-14151-s001.zip › ijms-1998230-supplementary.pdf]

Supplementary material

Activity and Synergy of Cu-ATCUN Antimicrobial Peptides

Jenna M. Greve and J. A. Cowan*

Department of Chemistry and Biochemistry, The Ohio State University, 100 West 18th Avenue, Columbus, Ohio 43210.

Table S1. Additive and indifferent FIC Values for OV3 derivatives with varying peptides in *E. coli* 25922.

A<br>B	OV3-00<br>OV3-01	Ov3-00<br>TRP-02	OV3-00<br>TRP-03	OV3-00<br>IND-01	OV3-00<br>Mag2-01	OV3-01<br>IND-01	OV3-01<br>TRP-02	OV3-01<br>TRP-03
[A]	1	2	0.5	2	1	1	2	1
MIC_A	2	4	2	2	1	4	8	8
[B]	2	0.25	8	32	1	32	4	16
MIC_B	4	8	16	32	1	32	8	16
**FIC**	**1.0**	**0.53**	**0.75**	**2**	**2**	**1.25**	**0.75**	**1.125**

FIC Index = [A]/[MIC_A] + [B]/[MIC_B], where [A] and [B] are the concentrations of respective peptides at which no growth was observed when dosed in combination, and [MIC_A] and [MIC_B] are the concentrations where no growth was observed when dosed separately.

Table S2. Additive and indifferent FIC values for CP10A and TempL with varying peptides in *E. coli* 25922.

A<br>B	CP10A<br>IND-01	CP10A<br>TRP-01	CP10A<br>TRP-02	CP10A<br>TRP-03	TempL<br>CP29-01	TempL<br>TRP-02	TempL<br>TRP-03
[A]	8	4	4	8	2	2	2
MIC_A	16	8	8	16	4	4	4
[B]	4	8	4	4	1	2	4
MIC_B	32	16	16	16	4	8	16
**FIC**	**0.625**	**1**	**0.75**	**0.75**	**0.75**	**0.75**	**0.75**

For Tables S3-S10: FIC Index = [A]/[MIC_A] + [B]/[MIC_B], where [A] and [B] are the concentrations of respective peptides at which no growth was observed when dosed in combination, and [MIC_A] and [MIC_B] are the concentrations where no growth was observed when dosed separately. The data from each experiment repeated in duplicate or triplicate is shown and bolded data in the final row is the average FIC value. Error is reported as the standard deviation of the mean.

Table S3. FIC values for Interactions of CP10A and TempL in *E. coli* 25922. * Previously reported data from reference [13] for comparison.

**A**<br>**B**	**CP10A**<br>**Sub5-01 ***	**CP10A**<br>**Mag2-01**	**CP10A**<br>**OV3-01**	**TempL**<br>**Sub5-01**	**TempL**<br>**TRP-01**	**TempL**<br>**IND-01**	**TempL**<br>**Mag2-01**	**TempL**<br>**OV3-01**
[A]	4/4/2	4/.25/4	0.5/2/.5	0.5/1/2	1/2/1	2/2	1/1/1	4/4/4
MIC_A	16/16/8	16/8/16	16/8/16	4/4/4	4/4/4	4/8	4/4/4	16/16/16
[B]	0.5/1/0.5	2/4/.25	1/0.5/1	4/4/4	8/0.25/4	0.25/4	4/4/.5	1/1/2
MIC_B	4/4/4	16/16/2	4/8/4	8/8/8	32/32/16	32/32	16/16/2	4/4/4
FIC	0.375/0.5/0.375	0.375/0.28/0.375	0.28/0.312/0.28	0.625/0.75/1	0.5/0.5/0.5	0.5/0.375	0.5/0.5/0.5	0.5/0.5/0.75
**FIC_{Avg}**	**0.42 ± 0.07**	**0.34 ± 0.05**	**0.29 ± 0.02**	**0.78 ± 0.2**	**0.5**	**0.44 ± 0.09**	**0.5**	**0.58 ± 0.14**

Table S4. FIC values for the non-ATCUN containing parental peptides in *E. coli* 25922.

A<br>B	CP10A<br>Sub5 [13]	CP10A<br>Mag2	CP10A<br>OV3-00	TempL<br>Sub5	TempL<br>TRP	TempL<br>Mag2	TempL<br>OV3-00
[A]	2/4/4	2/2/1	2/2/0.5	2/2/2	1/1/1	0.25/1/2	2/2/2
MIC_A	16/16/16	4/4/2	4/4/4	4/4/4	2/4/2	4/4/4	16/16/16
[B]	1/0.5/0.25	2/2/2	0.5/1/1	4/4/4	4/4/4	4/2/0.125	4/1/1
MIC_B	4/2/4	4/4/4	2/2/2	8/8/8	8/8/8	8/8/8	8/2/2
FIC	0.375/0.328/0.5	1/1/1	0.75/1/0.625	1/1/1	1/0.75/1	0.56/0.5/0.52	0.625/0.625/0.625
**FIC_{Avg}**	**0.40 ± 0.04**	**1**	**0.78 ± 0.2**	**1**	**0.9 ± 0.14**	**0.53 ± 0.03**	**0.625**

Table S5. FIC Values for ATCUN peptide derivatives in MRSA 43300.

A<br>B	CP10A<br>Sub5-01 *	CP10A<br>^Mag2-01	CP10A<br>OV3-01	TempL<br>Sub5-01	TempL<br>TRP-01	TempL<br>IND-01	TempL<br>^Mag2-01	TempL<br>OV3-01
[A]	0.125/0.125/0.25	1/1/1	.5/1/1	4/4/4	1/.5/1	.5/.25/.5	4/4/4	4/4/4
MIC_A	2/2/4	4/4/4	4/4/4	8/8/8	4/4/4	4/4/4	4/4/4	16/16/16
[B]	4/4/4	4/4/4	8/8/8	2/2/2	16/16/16	32/32/32	0.125/0.125/0.125	1/1/1
MIC_B	16/16/16	>64/>64/>64	32/32/32	4/4/4	32/32/32	64/64/64	>16/>16/>16	4/4/4
FIC	0.375/0.375/0.5	0.31/0.31/0.31	0.375/0.5/0.5	1/1/1	0.75/0.625/0.75	0.625/0.56/0.625	1/1/1	0.5/0.5/0.5
**FIC_{Avg}**	**0.42 ± 0.07**	**0.31**	**0.45 ± 0.07**	**1**	**0.7 ± 0.07**	**0.6 ± 0.04**	**1.0**	**0.5**

Table S6. FIC values for Parental Peptides in MRSA 43300. ^Mag2-01 has no detectable MIC in MRSA 43300, FIC value calculated from lowest detectable concentration off of FIC plate.

**A**<br>**B**	**CP10A**<br>**Sub5** [13]	**CP10A^**<br>**Mag2**	**CP10A**<br>**OV3-00**	**TempL**<br>**Sub5**	**TempL**<br>**TRP**	**TempL^**<br>**Mag2**	**TempL**<br>**OV3-00**
[A]	2/4/4	2/2/2	1/1/1	4/2/4	4/4/8	ND	4/4/4
MIC_A	8/16/16	2/4/4	4/4/4	8/8/8	8/8/8	ND	16/16/16
[B]	0.5/1/1	0.125/16/32	4/4/4	2/2/2	1/1/4	ND	0.5/0.5/.25
MIC_B	1/2/2	(>64)	8/8/8	4/4/4	4/4/4	ND	2/2/1
FIC	0.75/0.75/0.75	1/0.75/1	.75/0.75/0.75	1/.75/1	1/1/2	ND	0.5/0.5/0.5
**FIC_{Avg}**	**0.75**	**0.92**	**0.75**	**0.9 ± 0.1**	**1.3 ± 0.5**	**ND**	**0.5**

Table S7. FIC values for ATCUN derivatives with VRE. ** Biofilm Formation.

**A<br>B**	**CP10A<br>Mag2-01**	**CP10A<br>OV3-01**	**TempL<br>Sub5-01**	**TempL<br>TRP-01**	**TempL<br>IND-01**	**TempL**<br>Mag2-01**
[A]	1/1/1	1/1/1	ND	2/2/1	2/2/2	8/8/4
MIC_A	8/8/8	8/8/8	ND	4/4/4	4/4/4	8/8/4
[B]	4/4/4	0.5/0.5/0.5	ND	4/4/8	8/8/8	>32
MIC_B	64/64/64	8/8/8	ND	8/8/16	16/16/16	(>32)
FIC	0.19/0.19/0.19	. 0.19/0.19/0.19	ND	1/1/.75	1/1/1	**
**FIC_{Avg}**	**0.19**	**0.19**	**ND**	**0.92 ± 0.14**	**1.0**	**

Table S8. FIC values for Parental Peptides with VRE. ** Biofilm Formation.

**A<br>B**	**CP10A<br>Mag2**	**CP10A<br>OV3-00**	**TempL<br>Sub5**	**TempL<br>TRP**	**TempL**<br>Mag2**
[A]	1/1/1	1/1/1	ND	2/2/2	8/4/4
MIC_A	8/4/4	8/8/8	ND	4/4/4	8/8/4
[B]	2/4/2	1/1/0.5	ND	4/4/4	32/32/32
MIC_B	64/64/64	8/8/8	ND	8/8/8	32/32/32
FIC	0.28/0.31/0.28	0.25/0.25/0.19	ND	1/1/1	2/1.5/2
**FIC_{Avg}**	**0.29 ± 0.02**	**0.23 ± 0.03**	**ND**	**1.0**	**

Table S9. FIC Values for ATCUN Derivatives with *Acinetobacter baumannii*.

**A**<br>**B**	**CP10A**<br>**Sub5-01** [13]	**CP10A**<br>**Mag2-01**	**CP10A**<br>**OV3-01**	**TempL**<br>**Sub5-01**	**TempL**<br>**TRP-01**	**TempL**<br>**IND-01**	**TempL**<br>**Mag2-01**
[A]	1/2/2	4/4/4	1/.25/1	ND	2/2/2	2/2/2	2/2/2
MIC_A	4/4/8	16/16/16	4/4/4	ND	4/4/4	8/8/8	8/8/8
[B]	0.25/0.5/0.25	.25/.5/1	1/1/1	ND	8/8/8	8/8/8	2/2/2
MIC_B	1/2/1	4/4/4	4/4/4	ND	16/16/16	16/16/16	8/8/8
FIC	0.5/0.75/0.5	0.31/0.375/0.5	0.5/0.31/0.5	ND	1/1/1	0.75/.75/.75	0.5/0.5/0.5
$\mathbf{FIC_{Avg}}$	**0.58 ± 0.14**	**0.395 ± 0.09**	**0.44 ± 0.1**	**ND**	**1.0**	**0.75**	**0.5**

Table S10. FIC values for Parental Peptides with *Acinetobacter baumannii*.

**A**<br>**B**	**CP10A**<br>**Sub5** [13]	**CP10A**<br>**Mag2**	**CP10A**<br>**OV3-00**	**TempL**<br>**Sub5**	**TempL**<br>**TRP**	**TempL**<br>**Mag2**
[A]	2/2/2	4/4	2/2/1	ND	2/2/2	2/2/2
MIC_A	8/4/4	8/16	4/4/4	ND	4/4/4	4/4/4
[B]	0.5/0.5/0.5	0.5/1	1/.5/.5	ND	2/1/2	2/2/2
MIC_B	2/2/2	4/4	1/2/1	ND	8/8/8	8/8/8
FIC	0.5/0.75/0.75	0.56/0.5	1.5/.75/.75	ND	0.75/0.625/0.75	0.75/0.75/0.75
$\mathbf{FIC_{Avg}}$	**0.66 ± 0.14**	**0.53 ± 0.04**	**1.0 ± 0.43**	**ND**	**0.71 ± 0.07**	**0.75**
